# Reaction Behavior of Porous TiAl_3_ Intermetallics Fabricated by Thermal Explosion with Different Particle Sizes

**DOI:** 10.3390/ma14237417

**Published:** 2021-12-03

**Authors:** Kaiyang Li, Tiance Zhang, Yuanzhi Zhu

**Affiliations:** Department of Materials Science and Engineering, School of Mechanical and Materials Engineering, North China University of Technology, 5 Jinyuanzhuang Road, Beijing 100144, China; tokaiyang@163.com (K.L.); toztc1@126.com (T.Z.)

**Keywords:** porous TiAl_3_, intermetallics, thermal explosion, particle size, reaction behavior

## Abstract

Porous TiAl_3_ intermetallics were prepared by the thermal explosion (TE) and space holder method with different particle sizes of Ti and Al powders, and their reaction behaviors were investigated. The results showed that with the increase in the particle size of the Ti and Al powders, the interfacial contact between the particles decreased, resulting in low interfacial energy and reaction activity, making the process difficult to initiate. Meanwhile, the heat flow rose from 358.37 J/g to 730.17 J/g and 566.74 J/g due to the extension of the solid–liquid diffusion time. The TiAl_3_ structures obviously expanded, and the formation of connected pore channels promoted the porosity. Only when the Ti and Al particle sizes were both small did the solid–solid diffusion significantly appear. At the same time, the TE reaction weakened, so the product particles had no time to fully grow. This indicates that the particle size of the raw materials controlled the TE reaction process by changing the solid–liquid diffusion reaction time and the degree of solid-phase diffusion.

## 1. Introduction

Porous materials refer to a network material of interconnected or closed voids that can control the shape, size, and uniformity of porous space. A pore boundary is composed of a columnar or planar structure, which has special dual characteristics of function and structure, and it is regarded as a new type of structural and functional integrated material [1,2,3]. In recent years, porous intermetallic compounds such as Ti-Al, Ni-Al, and Nb-Al have been developed rapidly due to their advantages as porous metals and porous ceramics [4,5,6]. Ti-Al intermetallic compounds (α2-Ti_3_Al, γ-TiAl, and TiAl_3_) are the most widely studied porous intermetallic compounds. Compared with γ-TiAl and α2-Ti_3_Al, TiAl_3_ has the highest specific modulus (220 GPa), the lowest density (3.4 g/cm^3^), the best high-temperature oxidation resistance, and an open pore structure [7,8]. In addition, TiAl_3_ can be used as a porous structure material due to the formation of open connected pore structures during sintering. Therefore, TiAl_3_ is accepted as the best porous material because of the excellent properties mentioned above and could be well applied in thermal insulation, filtration, catalytic conversion, and other industrial fields [9,10,11,12,13].

Combustion synthesis (CS), including self-propagating high-temperature synthesis (SHS) and thermal explosion (TE), has attracted the attention of a large number of researchers due to its advantages of rapid reaction, simple conditions, and time savings [5,14]. SHS is a process of heating one end of the reactant compact and promoting the continuation of self-reaction by virtue of the heat released during the reaction between materials [15,16]. Shen et al. [17] prepared materials by SHS, staged sintering, and found that the compact prepared by SHS was prone to cracking due to uneven heating. However, thermal explosion (TE) heats the compact as a whole, and when it reaches the ignition point, a rapid exothermic reaction occurs. The temperature rises sharply in a short time, and the whole process is similar to an explosion. TE has the strengths of a fast reaction and low energy consumption, and it can effectively avoid cracking in the sintering process. It is considered an excellent mode to fabricate Ti-Al intermetallic compounds [18,19,20]. Xiong et al. [21] first reported that the combustion reaction of the Ti-Al intermetallic compounds was related to the powder particle size of the raw materials. Jiang et al. [11,22] explained that the Kirkendall effect controlled the pore formation of the porous Ti-Al intermetallic compounds by solid–solid diffusion, and the powder particle size directly affected its average pore size. Nevertheless, in the sintering process of the Ti-Al intermetallic compound, the occurrence of TE requires the presence of liquid Al, and the pore-forming mechanism was no longer the Kirkendall effect but intense solid–liquid diffusion [23,24]. Jiao et al. [18,24,25,26,27] studied the pore-forming process under the thermal explosion reaction and successfully manufactured a porous TiAl_3_ intermetallic compound by adding space holders. Che et al. [28] showed that the reaction mechanism under solid–liquid diffusion was connected with the diffusion rate and diffusion distance of Ti and Al. This means that the particle size of the powder probably impacts the reaction process under solid–liquid diffusion. Following the insights of previous work, the influence of particle sizes on the reaction behavior of material processed by thermal explosion in combination with NaCl space holders are quantified. Understanding the reaction behavior of porous TiAl_3_ in TE mode under different particle sizes can provide a new approach for the preparation of porous TiAl_3_ intermetallic compounds.

In the present work, porous TiAl_3_ intermetallics were prepared by Ti-75%Al introducing 60% NaCl space holders, with three different average initial powder sizes (10, 40, and 70 μm). The thermal response, phase composition, expansion behavior, and product morphology of the alloy were studied. Importantly, the influence of the powder particle size on the reaction process of TiAl_3_ synthesis by TE was expounded and provided a theoretical basis for selecting raw materials in the preparation process of TiAl_3_, according to various industrial demands.

## 2. Materials and Methods

The elements Ti (99.9% purity) and Al (99.9% purity) with different particle sizes (10, 40, and 70 μm) were mixed in a planetary ball mill at a ratio of 1:3. The ball milling process took place in a ball milling tank containing alumina balls. Before adding powder, the ball milling tank and alumina balls were cleaned with alcohol and blow-dried to avoid pollution in the experiment. After completion, a 60% volume ratio of NaCl (99.9% purity) space holders was added to blend with the dried Ti-75%Al powder. The particle size of NaCl ranged from 200 to 400 μm. Then, the mixture was cold pressed in the powder tablet press at a pressure of 300 MPa to form cylindrical green compacts with a diameter of 14 mm and height of 4 mm. The green compacts were dissolved in room temperature water for 64 h to completely remove the NaCl space holder. Subsequently, the green compacts were placed in a vacuum sintering furnace (pressure 10^−3^ MPa) and were kept for 30 min after heating to 700 °C at a heating rate of 15 °C/min, and then the furnace cooled to the room temperature.

In order to evaluate the heat absorption and release process in the sintering process, differential scanning calorimetry (DSC) was used to measure the heat absorption and release of the sintered discs with a heating rate of 15 °C/min from 20 to 800 °C. The phase composition of the sintered discs was determined by X-ray diffraction (TD-3500, Tongda, Liaoning, China) with Cu Kα radiation operated at a voltage of 30 kV, a tube current of 20 mA, step size of 0.04°, and a scanning rate of 10°/min. In order to describe the change in the expansion ratio, expansion ratio tests were carried out five times on the samples before and after sintering. The porosity was measured according to Archimede’s principle. The mercury injection method was used to determine the average micropore size. A Scanning Electron Microscope, SEM (Sigma-300, ZEISS, Oberkochen, Germany), was used to examine the morphology of the produced samples. 

## 3. Results and Discussion

### 3.1. Reaction Process

**Figure 1:**
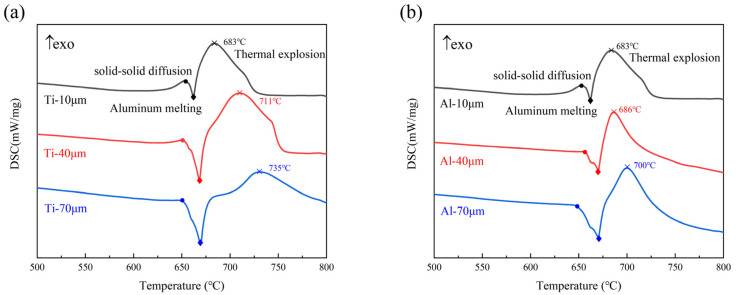
DSC results of samples under different titanium and aluminum particle sizes (Diamonds represent solid–solid exothermic peaks, circles represent Al melting endothermic peaks, and crosses represent the thermal explosion exothermic peaks): (**a**): aluminum particle size 10 μm with different titanium particle sizes, (**b**): titanium particle size 10 μm with different aluminum particle sizes.

Figure 1a,b shows the DSC results of adding a different powder size for titanium or aluminum when the size of the other powder was 10 μm, revealing the thermal response of the entire sintering process at the heating rate of 15 °C/min. No matter how the particle size of the powder changed, there were two exothermic peaks and one endothermic peak in the DSC curve. The first mild exothermic peak occurred at temperatures slightly below the melting point of aluminum, illustrating a weak solid–solid diffusion reaction prior to aluminum melting due to the difference in the diffusion coefficients of the two elements [29]. Subsequently, a slight endothermic peak appeared near the melting point temperature of aluminum, representing the beginning of aluminum melting. The last sharp exothermic peak corresponded to the thermal explosion. At this time, the aluminum powder completely melted and rapidly diffused around the Ti powder, resulting in a violent solid–liquid diffusion thermal explosion reaction to form the TiAl_3_ intermetallic compounds and released a large amount of heat. These observations fit the description provided by Jiao et al. [24,30]. Peng et al. [31] confirmed that solid titanium and liquid aluminum would have a strong heating reaction, and the reaction rate was swift.

**Figure 2:**
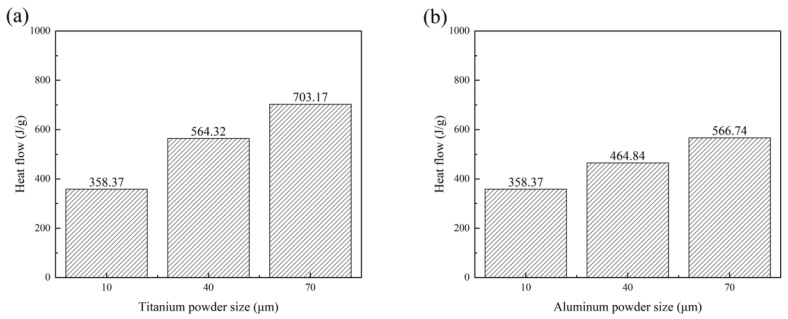
Heat flow of samples with different titanium and aluminum particle sizes: (**a**): aluminum particle size 10 μm with different titanium particle sizes, (**b**): titanium particle size 10 μm with different aluminum particle sizes.

As shown in Figure 1a,b, with the increase in powder particle size, the exothermic peaks of the thermal detonation reaction of Ti and Al migrated to higher temperatures (from 683 °C to 735 °C and 700 °C, respectively), and the exothermic peaks widened significantly. By calculating the area of the exothermic peak of the solid–liquid diffusion reaction in the DSC curves, the heat flow increased from 358.37 to 703.17 J/g and 566.74 J/g with the increase in the powder particle size, as shown in Figure 2. In addition, when the Al particles were large, the exothermic peak of the solid–solid diffusion was almost invisible. Only when the Ti and Al particles were small did a relatively obvious solid–solid diffusion exothermic peak appear. Both large Al or Ti particles reduced the interface contact between particles, and the interface energy and reaction activity were low, which required a strong diffusion driving force at the higher temperature, and the exothermic peak shifted to a higher temperature, increasing the difficulty of the thermal explosion reaction [21]. DSC results of both particles increased from 10 μm to 70 μm are shown in the Supplementary Figure S1. It can be seen from the figure that when both particles increased to 40 and 70 μm, there was also no obvious solid–solid diffusion. This also indicated that the larger particle size caused the reaction change rather than the difference between particles.

As Al is the main diffusion component in the Ti-Al binary diffusion system, when the temperature rose to the melting point of Al, the excellent wettability of Ti enabled the liquid Al to gradually spread inward on the edge of Ti particles under the action of surface tension, until the Ti particles were completely wrapped, and TiAl_3_ was formed [28,32]. The large Ti or Al particles increased the distance of the solid–liquid diffusion and prolonged the diffusion time, which led to the widening of the exothermic peak and the elevation of the heat flow. Due to the existence of pores between particles in the cold pressing process and pores formed by the NaCl space holder, the pressure difference on the pore surface was the driving force of liquid Al diffusion [33]. Assuming that Al melted completely and the Ti particles were completely spherical, the pressure difference P of liquid phase spreading can be obtained as follows [33]:

(1) $$P=\frac{2\gamma }{r}$$

where γ is the surface tension, and r is the particle size of Ti. It can be clearly seen from the above equation that with the increase in Ti particle size, the pressure difference decreases and the reaction speed declines, which further prolongs the diffusion time and results in the continuous generation and accumulation of heat in the reaction process, causing significant heat flow. Because the diffusion rate of Al in Ti is higher than that of Ti in Al, the contact area between Al and small Ti particles is large, which makes it easier to diffuse to the Ti surface and conducive to the solid-phase diffusion. This means that the content of Al in the liquid–phase diffusion process is reduced, resulting in lower heat flow. The increase in Ti particle size also extends the diffusion distance and reduces the solid phase diffusion effect. Therefore, only when the particle size of both Ti and Al is small can the solid phase diffusion be obvious.

**Figure 3:**
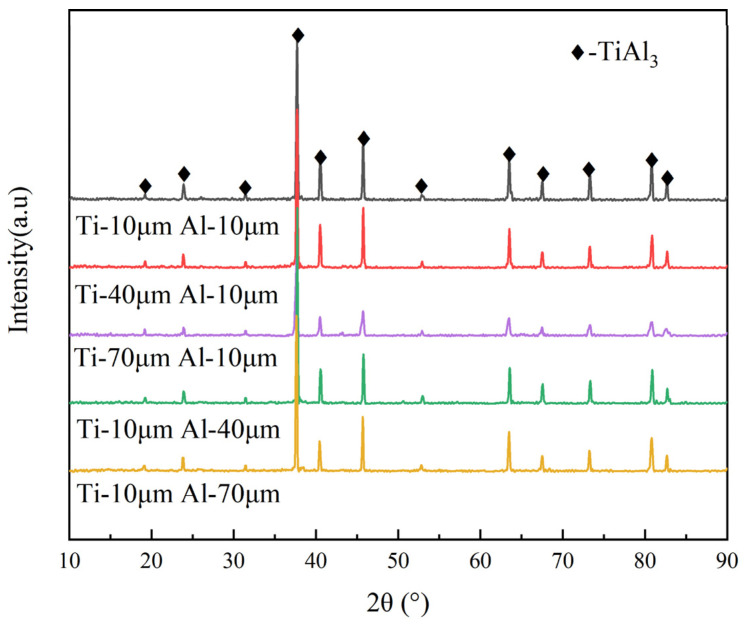
XRD pattern of samples with different titanium and aluminum particle sizes.

Figure 3 shows the XRD patterns of sintered samples with different particle sizes. Only a TiAl_3_ peak was found in the XRD pattern. This illustrates that no matter how the Ti or Al particle size changed, there was no significant effect on phase composition. Although the increase in particle size makes the reaction harder to initiate, the thermal explosion reaction does fully finish, and all the products are converted into the TiAl_3_ phase under sufficient holding time [30]. In addition, no NaCl peak was found in the sintered sample, proving that the original NaCl had been completely dissolved.

### 3.2. Expansion Behaviors and Porosity

**Figure 4:**
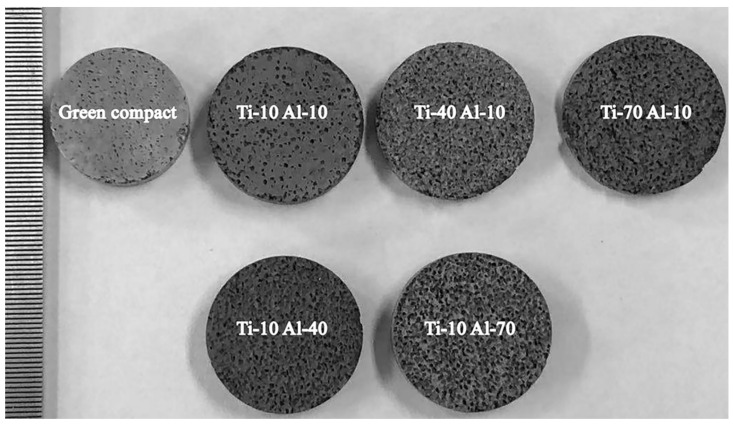
The macroscopic morphology of green compact and sintered discs under different titanium and aluminum particle sizes.

**Figure 5:**
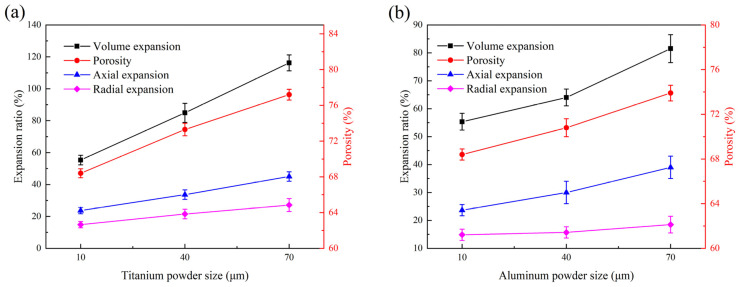
Expansion ratio and porosity of samples with different titanium and aluminum particle sizes: (**a**): aluminum particle size 10 μm with different titanium particle sizes, (**b**): titanium particle size 10 μm with different aluminum particle sizes.

Figure 4 depicts the macroscopic morphology of samples. The structure of samples had no damage and showed a porous network. It is worth mentioning that the specimen after sintering was obviously expanded compared with that before sintering, and the corresponding expansion ratio is shown in Figure 5. Expansion was related to heat release and volume exchange contraction during TiAl3 production. In the process of the thermal explosion reaction, volume exchange in the transformation of Ti + 3Al → TiAl_3_ generates the contraction of the molar volume of TiAl_3_ system [34]. However, a large amount of reaction heat is accumulated in the TE reaction process, and the sample is difficult to completely dissipate to cause thermal expansion. On the other hand, after the TE reaction, the pore size increases after the dissolution of the space holder, and the residual air inside the pore of the space holder expands after heating, further promoting the expansion of sintered discs. Similar behaviors were also reported by Liang et al. [32] The expansion caused by the above reasons is much higher than the volume contraction of the formation of TiAl_3_ structure, so the sintered discs show expansion.

The variations in the volume expansion ratio, radial expansion ratio, axial expansion ratio, and porosity of TiAl_3_ sintered discs with different powder particle sizes are shown in Figure 5. It can be observed from the figure that with the increase in particle size of Ti or Al, the variation of the volume expansion ratio of TiAl_3_ is consistent with the trend of reaction heat flow, shown in Figure 2, which increased from 55.32% to 116.21% and 81.51%, respectively. In fact, large particles of Ti or Al powder accumulate a lot of heat during the reaction process, which enables the latent heat release to be significantly increased. Nevertheless, the specific surface energy of the small particle raw powder in the reaction process is higher, and the specific surface energy is the driving force of shrinkage of the TiAl_3_ structure in the later stage of the sintering process [22]. The reduction in the shrinkage driving force and the release of more heat energy enable the expansion caused by large particles to be more prominent. Furthermore, the porosity and expansion behavior variation were also very similar, which increased from 68.4% to 77.2% and 73.9%. Large particle powders had hardly any solid-phase diffusion, and more liquid Al participated in the reaction. The flow of liquid Al produces a large number of connected pore channels in the TE process, which significantly increases the porosity [35]. High heat flow and intense expansion also cause the internal pores to grow and connect, leading to the higher porosity of the large particle powder samples.

### 3.3. Microstructure Evolution

**Figure 6:**
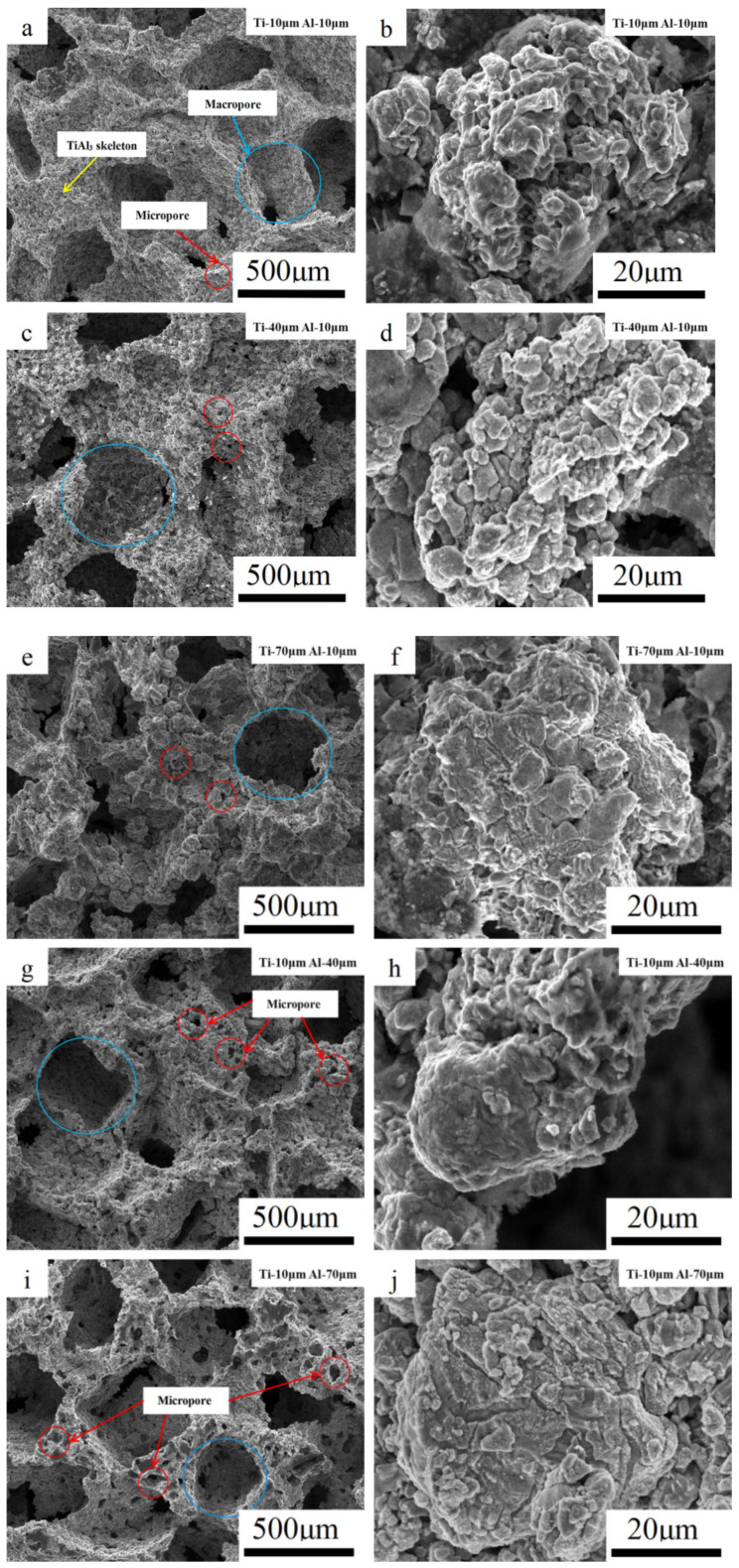
SEM images of the TiAl_3_ microstructure and product particle morphology: (**a**,**b**): Ti-10 μm–Al-10 μm, (**c**,**d**): Ti-40 μm–Al-10 μm, (**e**,**f**): Ti-70 μm–Al-10 μm, (**g**,**h**): Ti-10 μm–Al-40 μm, and (**i**,**j**): Ti-10 μm–Al-70 μm.

The microstructure of the porous TiAl_3_ intermetallics is shown in Figure 6. Regardless of the particle size of Ti or Al, the main structure of the TiAl_3_ remained unchanged, consisting of a TiAl_3_ skeleton, macropores left after the dissolution of the NaCl space holder, and micropores generated after intense solid–liquid diffusion. Figure 6b,d,f,h,j shows the morphologies of the product particles with different powder particle sizes. With the increase in the powder particle sizes, the connection of the product particles gradually became closer. According to the change of heat flow, as shown in Figure 2, it can be analyzed that the TE reaction needs continuous heat release, and the product particles grow completely with the heat release accumulated by the large particle powder sample in the sintering process. Although an obvious TE process occurred in the short time of the exothermic reaction of the small particle powder samples, the significant solid–solid diffusion consumed part of the Al before the occurrence of the TE, resulting in no time for further growth and cooling of product particles in the TE process [26].

**Figure 7:**
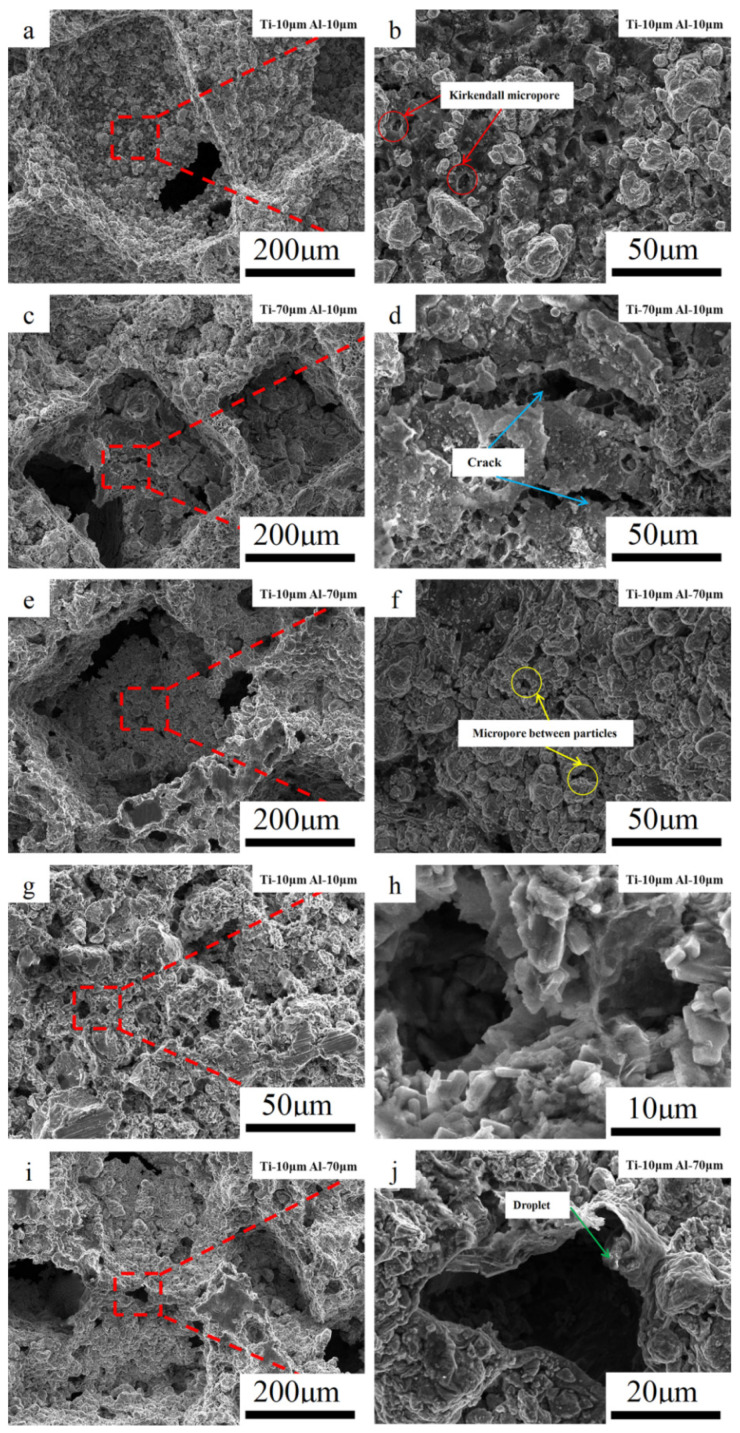
SEM images of the internal microstructure of TiAl_3._ (**a**–**f**) shows the pore wall morphology formed by NaCl, (**a**,**b**): Ti-10 μm–Al-10 μm, (**c**,**d**): Ti-70 μm–Al-10 μm, and (**e**,**f**): Ti-10 μm–Al-70 μm; (**g**–**j**) represents the micropore morphology of samples’ skeleton with different Al particle sizes, (**g**,**h**): Ti-10 μm–Al-10 μm, and (**i**,**j**): Ti-10 μm–Al-70 μm).

**Figure 8:**
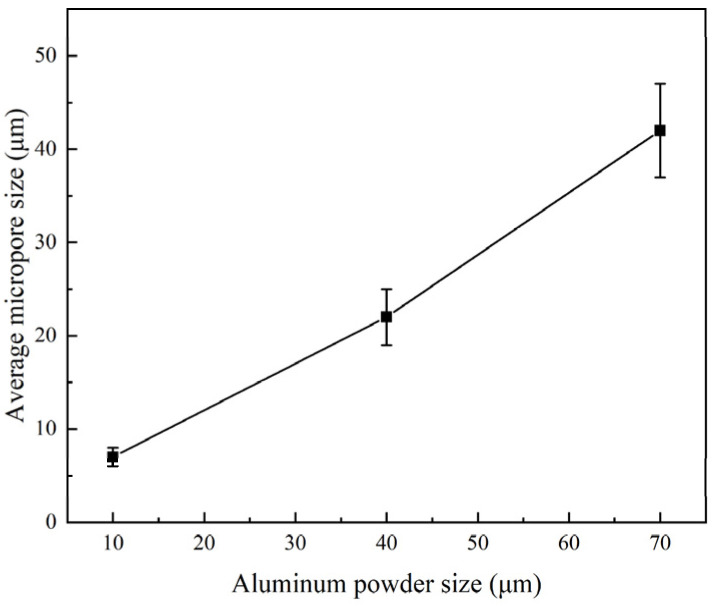
Average micropore size of samples with different Al particle sizes.

Figure 7a–f shows the pore wall morphology inside the pores formed by NaCl. Kirkendall micropores were obviously seen in the sintered TiAl_3_ discs with small powder particle sizes. Jiang et al. [6] proved that Kirkendall micropores are formed by solid–solid diffusion. Jiao et al. [24,25] explained that the TE reaction pore-forming mechanism was no longer the Kirkendall effect. This indicates that the sample with small particle sizes underwent partial solid–solid diffusion before the TE reaction occurred. However, only the micropores between product particles were found in the pore structure formed by NaCl in the large Al particle sample, and no cracks or Kirkendall micropores were found. In addition, cracks appeared in the pore wall of the samples with the larger Ti particle size, and the pore wall was divided into small islands shaped by the cracks. According to Figure 2 and Figure 5, the large particle Ti sample had severe heat release and significant expansion, concluding that the external expansion of the pore wall under heat, which led to tensile stress on the surface of the pore wall. The volume exchange generated by TiAl_3_ resulted in compressive stress on the surface of the TiAl_3_ particles. The large particle Ti caused excessive expansion in the diffusion process, large enough residual stress to cause the crack. [34]. The formation of such a cracked structure assists in the communication of large pores with other internal pores and improves porosity. Figure 7g–j is the skeleton of the TiAl_3_ with different Al particle sizes and the micropores formed after TE reaction. It can be seen that with the increase in the Al particle size, the micropore size also increases significantly. Figure 8 shows the change in average micropore size with Al particle size; when the Al particle size increases from 10 μm to 70 μm, the average micropore size increases from 7 μm to 42 μm.

**Figure 9:**
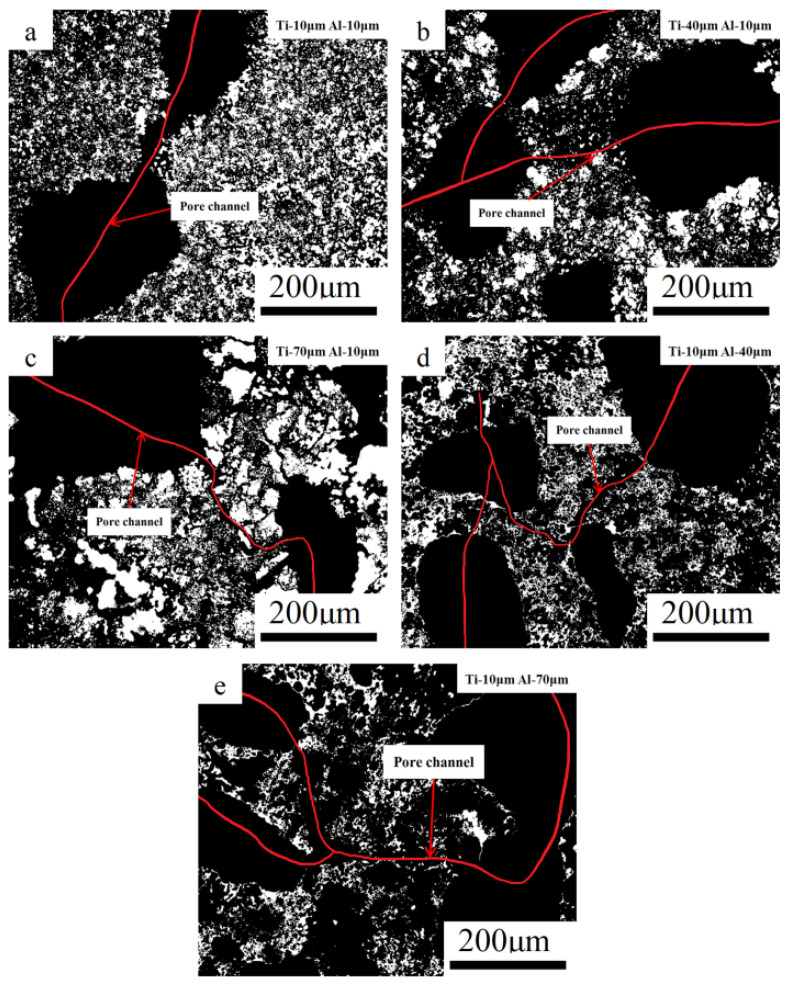
Optical microscope images of the skeleton and pores of the TiAl_3_ sintered discs with different particle sizes: (**a**): Ti-10 μm–Al-10 μm, (**b**): Ti-40 μm–Al-10 μm, (**c**): Ti-70 μm–Al-10 μm, (**d**): Ti-10 μm–Al-40 μm, and (**e**): Ti-10 μm–Al-70 μm.

Therefore, combining Figure 7 and Figure 8, one can draw the conclusion that the micropores formed by the thermal explosion are positively correlated with Al particle size. Traces of molten droplets can be seen at the edges of the pores in Figure 7j. The TE reaction occurred after Al melting, the Ti particles were gradually wrapped by the melted Al liquid, the Ti particles were gradually dissolved in the Al liquid, and TiAl_3_ was precipitated in the saturated Ti-Al solution. Micropores appeared at the initial position of Al melting, and the original morphology of Al particles remained. This resulted in an increase in average micropore size and porosity. The optical microscopic metallographies with different particle sizes are shown in Figure 9, in which the micropores and cracks formed in the structure can be intuitively seen. The pores formed by NaCl are connected with other pores by cracks or micropores, which increases the porosity of the system and forms a series of connected pore channels.

## 4. Conclusions

Porous TiAl_3_ intermetallics were successfully fabricated by the thermal explosion method with 60% NaCl space holders at different powder sizes (10, 40, and 70 μm). The final products were transformed into the TiAl_3_ phase, and TiAl_3_ maintained a porous network structure with NaCl macropores, micropores, and pore channels. With the increase in particle size, the interfacial contact between particles decreased, the reaction diffusion distance of TE increased, and the diffusion time became longer. The exothermic peak migrated to a higher temperature (683 °C to 735 °C and 700 °C, respectively), raising the reaction difficulty. The latent heat of the reaction release increased from 358.37 J/g to 730.17 J/g and 566.74 J/g, respectively. Sufficient exothermic heat and long reaction time caused the final product particles to grow completely. Only when both Ti and Al had a small particle size was the solid phase diffusion obvious before the TE. The TiAl_3_ structure formed by large Ti particles had the highest expansion ratio (116.21%) and porosity (77.2%), and cracks occurred on the wall of the NaCl pore. The micropore size of the TiAl_3_ skeleton after TE reaction was positively correlated with the Al particle size. Both cracks and micropores formed interconnected pore channels and improved porosity. This provides a new understanding of the TE reaction process.

## Data Availability

Not applicable.

## Supplementary Materials

The following are available online at https://www.mdpi.com/article/10.3390/ma14237417/s1, Figure S1: DSC results of both particles increased from 10 μm to 70 μm.
