# Supplementary material for: Reaction Behavior of Porous TiAl3 Intermetallics Fabricated by Thermal Explosion with Different Particle Sizes
Source: Materials (Basel). 2021 Dec 3;14(23):7417. doi: 10.3390/ma14237417 (PMC8658783; doi:10.3390/ma14237417)
Supplement: Supplementary file 1 [file materials-14-07417-s001.zip › materials-1442369-supplementary.pdf]

materials

Supplementary Information

Reaction Behavior of Porous $TiAl_3$ Intermetallics Fabricated by Thermal Explosion with Different Particle Sizes

Kaiyang Li, Tiance Zhang and Yuanzhi Zhu *

Department of Materials Science and Engineering, School of Mechanical and Materials Engineering, North China University of Technology, 5 Jinyuanzhuang Road, Beijing 100144, China; tokaiyang@163.com (K.L.); toztc1@126.com (T.Z.)
* Correspondence: tozyz1@126.com; Tel.: +86-18610088415

Citation: Li, K.; Zhang, T.; Zhu, Y. Reaction Behavior of Porous $TiAl_3$ Intermetallics Fabricated by Thermal Explosion with Different Particle Sizes. *Materials* **2021**, *14*, 7417. https://doi.org/10.3390/ma14237417

Academic Editor: Filippo Berto Abílio M.P. De Jesus and José A.F.O. Correia

Received: 14 October 2021
Accepted: 27 November 2021
Published: 3 December 2021

Publisher's Note: MDPI stays neutral with regard to jurisdictional claims in published maps and institutional affiliations.

Copyright: © 2021 by the authors. Submitted for possible open access publication under the terms and conditions of the Creative Commons Attribution (CC BY) license (http://creativecommons.org/licenses/by/4.0/).

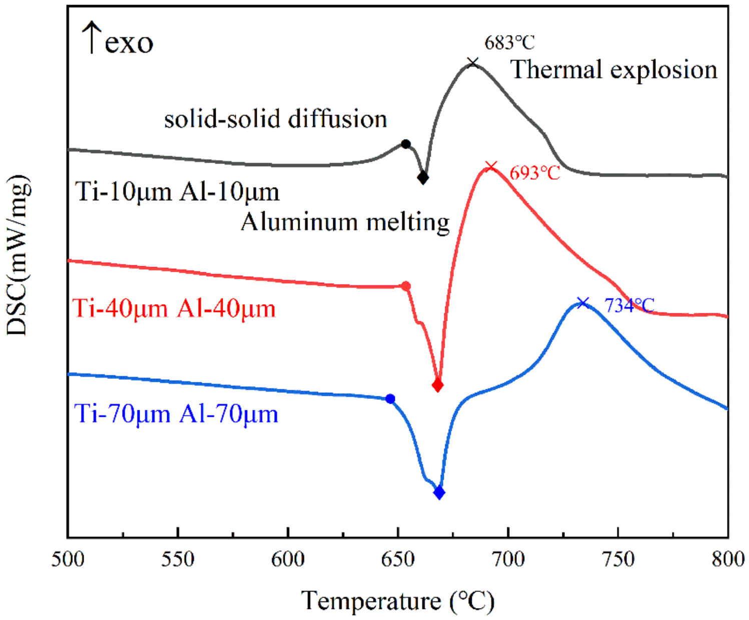

Figure S1. DSC results of both particles increased from 10 μm to 70 μm.
